# N-glycosylation in *Haloferax volcanii*: adjusting the sweetness

**DOI:** 10.3389/fmicb.2013.00403

**Published:** 2013-12-24

**Authors:** Jerry Eichler, Adi Arbiv, Chen Cohen-Rosenzweig, Lina Kaminski, Lina Kandiba, Zvia Konrad

**Affiliations:** Department of Life Sciences, Ben Gurion University of the NegevBeersheva, Israel

**Keywords:** Archaea, *Haloferax volcanii*, N-glycosylation, post-translational modification, protein glycosylation, S-layer glycoprotein

## Abstract

Long believed to be restricted to Eukarya, it is now known that cells of all three domains of life perform N-glycosylation, the covalent attachment of glycans to select target protein asparagine residues. Still, it is only in the last decade that pathways of N-glycosylation in Archaea have been delineated. In the haloarchaeon *Haloferax volcanii*, a series of Agl (*a*rchaeal *gl*ycosylation) proteins is responsible for the addition of an N-linked pentasaccharide to modified proteins, including the surface (S)-layer glycoprotein, the sole component of the surface layer surrounding the cell. The S-layer glycoprotein N-linked glycosylation profile changes, however, as a function of surrounding salinity. Upon growth at different salt concentrations, the S-layer glycoprotein is either decorated by the N-linked pentasaccharide introduced above or by both this pentasaccharide as well as a tetrasaccharide of distinct composition. Recent efforts have identified Agl5–Agl15 as components of a second *Hfx. volcanii* N-glycosylation pathway responsible for generating the tetrasaccharide attached to S-layer glycoprotein when growth occurs in 1.75 M but not 3.4 M NaCl-containing medium.

To cope with the challenges associated with life in a hypersaline environment, halophilic Archaea like *Haloferax volcanii* rely on a variety of strategies manifested at the molecular level. For instance, haloarchaeal proteins present more acidic residues and fewer basic residues than do their non-halophilic homologs (Lanyi, 1974; Fukuchi et al., 2003). While this approach allows haloarchaeal proteins to fold and function properly in the presence of molar concentrations of salt, modified amino acid composition does not allow such proteins to adapt to fluctuations in their surroundings. Instead, post-translational modifications offer proteins a route through which to respond to changing conditions in a transient manner. In the case of the *Hfx. volcanii* S-layer glycoprotein, the sole component of the protein shell surrounding the cell (Sumper et al., 1990), changes in environmental salinity are reflected in a modified N-glycosylation profile (Guan et al., 2012).

The *Hfx. volcanii* S-layer glycoprotein contains seven putative sites of N-glycosylation (Sumper et al., 1990), at least two of which are modified by a pentasaccharide comprising a hexose, two hexuronic acids, a methyl ester of hexuronic acid, and a mannose (Abu-Qarn et al., 2007; Guan et al., 2010; Magidovich et al., 2010). Genetic and biochemical approaches have served to identify a series of Agl (*a*rchaeal *gl*ycosylation) proteins responsible for the assembly and attachment of this N-linked glycan. AglJ, AglG, AglI, and AlgE are glycosyltransferases that sequentially add the first four sugars of the N-linked pentasaccharide to a common dolichol phosphate carrier (Abu-Qarn et al., 2008; Yurist-Doutsch et al., 2008; Guan et al., 2010; Kaminski et al., 2010). Once the lipid-linked tetrasaccharide (and its precursors) has been “flipped” across the plasma membrane, the glycan is delivered to the S-layer glycoprotein Asn-13 and Asn-83 positions by AglB, an oligosaccharyltransferase (Abu-Qarn et al., 2007). The final pentasaccharide sugar, mannose, is added to a distinct dolichol phosphate carrier on the cytoplasmic face of the membrane by the glycosyltransferase AglD, delivered across the membrane to face the cell exterior in a process involving AglR, and then transferred to the Asn-linked tetrasaccharide by AglS (Plavner and Eichler, 2008; Guan et al., 2010; Calo et al., 2011; Cohen-Rosenzweig et al., 2012; Kaminski et al., 2012). In addition, other Agl proteins serve various sugar-processing or other roles that contribute to pentasaccharide assembly, such as AglF, a glucose-1-phosphate uridyltransferase, AglM, a UDP-glucose dehydrogenase, AglP, a methyltransferase, and AglQ, an isomerase (Yurist-Doutsch et al., 2008; Magidovich et al., 2010; Yurist-Doutsch et al., 2010; Arbiv et al., 2013). The most recent version of the Agl pathway is presented in **Figure 1**.

**FIGURE 1:**
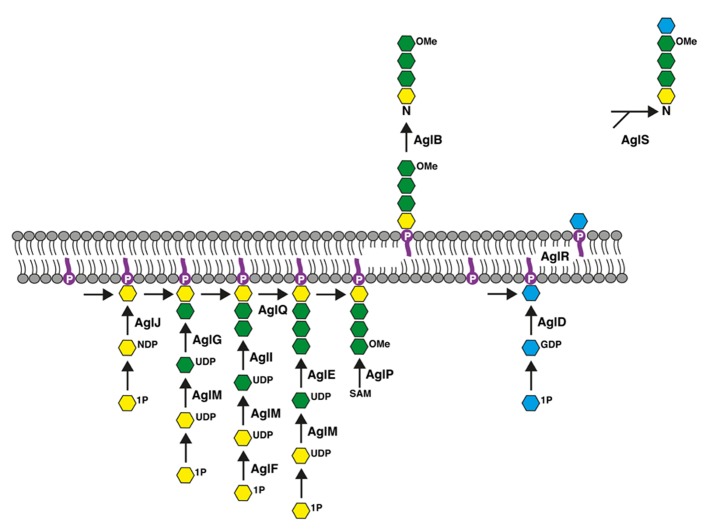
**Schematic depiction of the *Hfx. volcanii* Agl pathway used for the assembly and attachment of the pentasaccharide N-linked to the S-layer glycoprotein Asn-13 and Asn-83 positions.** See the text for details. In the figure, dolichol phosphate is in purple, hexose is in yellow, hexuronic acid is in green, mannose is in blue and OMe corresponds to a methyl ester group. The bottom half of the figure corresponds to the cell interior.

When first described, *Hfx. volcanii* was reported to grow at NaCl concentrations ranging from 1 M to over 4 M (Mullakhanbhai and Larsen, 1975). In deciphering the *Hfx. volcanii* pathway responsible for the assembly and attachment of the N-linked pentasaccharide decorating S-layer glycoprotein Asn-13 and Asn-83 delineated above, cells were grown in medium containing 3.4 M NaCl. However, when the S-layer glycoprotein was considered in cells grown in medium containing only 1.75 M NaCl, a different N-glycosylation profile was observed. When grown at the lower salinity, S-layer glycoprotein Asn-13 and Asn-83 were still modified by the pentasaccharide described above, although to a lesser extent than when the same cells were grown in 3.4 M NaCl-containing medium. What was more striking was that Asn-498, a position not modified when growth occurs at the higher salinity, was decorated by a novel “low salt” tetrasaccharide comprising a sulfated hexose, two hexoses and a rhamnose when cells were raised at the lower salinity (Guan et al., 2012). Moreover, the same tetrasaccharide was detected on dolichol phosphate in cells raised in 1.75 M NaCl-containing medium. Indeed, dolichol phosphate bearing the low salt tetrasaccharide had been previously reported when *Hfx. volcanii *cells were grown in medium containing only 1.25 M NaCl (Kuntz et al., 1997). Thus, both dolichol phosphate and the S-layer glycoprotein present bound glycans that differ as a function of growth medium salinity. Furthermore, medium salinity also dictated whether N-glycosylation sites in the S-layer glycoprotein were processed and to what extent. The finding that the *Hfx. volcanii *S-layer glycoprotein can be simultaneously modified by two very different N-linked glycans had also been reported to be true in a second haloarchaeon, namely *Halobacterium salinarum*. In work conducted some 30 years ago, it was reported that the S-layer glycoprotein in this organism is also modified by two distinct N-linked glycans (for a review, see Lechner and Wieland, 1989). However, unlike the situation in *Hbt. salinarum*, where relatively little is known of the pathway(s) recruited for N-glycosylation, work in the last decade has provided considerable insight into this post-translational modification in *Hfx. volcanii*, including the recently solved pathway of low salt tetrasaccharide assembly.

By combining gene deletions with mass spectrometric analysis of glycan-charged dolichol phosphate and S-layer glycoprotein-derived peptides, it was demonstrated that the Agl proteins responsible for assembly of the N-linked pentasaccharide are not involved in the biosynthesis of the low salt tetrasaccharide (Kaminski et al., 2013a). As such, efforts were directed at identifying genes encoding proteins comprising a second N-glycosylation pathway. Delineating components of the pathway responsible for generating the low salt tetrasaccharide initially relied on previous work showing that all of the *Hfx. volcanii* genes involved in the assembly of the N-linked pentasaccharide decorating S-layer glycoprotein Asn-13 and Asn-83, with the exception of *aglD*, are found in a single cluster spanning *HVO_1517 *(*aglJ*) to *HVO_1531 *(*aglM*; Yurist-Doutsch and Eichler, 2009; Yurist-Doutsch et al., 2010). As such, the *Hfx. volcanii* genome sequence (Hartman et al., 2010) was scanned for clustered open reading frames (ORFs) annotated as serving some glycosylation-related roles. Those ORFs spanning the region from *HVO_2046 *to *HVO_2061* represent one such cluster. The involvement of the products of *HVO_2046 *to *HVO_2061* in the biogenesis of the low salt tetrasaccharide was subsequently confirmed in a series of experiments involving gene deletions combined with mass spectrometry-based examination of dolichol phosphate and the S-layer glycoprotein. Given their roles in N-glycosylation, these proteins were re-annotated as Agl5–Agl15 (Kaminski et al., 2013a).

Based on the effects of *agl5*–*agl15* deletion on dolichol phosphate and S-layer glycoprotein Asn-498 glycosylation, together with the results of a bioinformatics-based examination of the encoded proteins, a model of the pathway responsible for low salt tetrasaccharide biogenesis has been proposed (Kaminski et al., 2013a; **Figure 2**). In this working model, Agl5 and Agl6 are implicated in adding the linking hexose to dolichol phosphate, while Agl7 contributes to the sulfation of this lipid-linked sugar. That population of dolichol phosphate-hexose seen in cells lacking either Agl5 or Agl6 likely corresponds to the lipid carrier charged with the first sugar of the pentasaccharide transferred to Asn-13 and Asn-83, a process that also occurs in low salt conditions (Guan et al., 2012). Furthermore, because cells lacking Agl7 contain dolichol phosphate charged with a non-sulfated version of the low salt tetrasaccharide, whereas no Asn-498-fused low salt tetrasaccharide (or its di- or tri-saccharide precursors) were detected in such cells, sulfation of the dolichol phosphate-bound hexose may be required for translocation of dolichol phosphate charged with a more elaborate low salt tetrasaccharide precursor or the complete glycan itself across the plasma membrane. Clearly, additional studies are needed to precisely define the actions of Agl5, Agl6, and Agl7, as well as their order of action. While the enzyme responsible for adding the second sugar of the low salt tetrasaccharide, a hexose, to sulfated hexose-charged dolichol phosphate remains to be identified, it appears that Agl8 and Agl9 contribute to the addition of the next sugar, a hexose, to disaccharide-charged dolichol phosphate. Agl10–14 are involved in the subsequent appearance of a rhamnose to the dolichol phosphate-bound trisaccharide, yielding the complete low salt tetrasaccharide on the lipid carrier. In cells lacking Agl15, the intact low salt tetrasaccharide is assembled on dolichol phosphate but no such glycan is detected on S-layer glycoprotein Asn-498. This observation is consistent with Agl15 serving as a flippase, mediating the translocation of low salt tetrasaccharide-charged dolichol phosphate (and likely dolichol phosphate bearing tetrasaccharide precursors) across the membrane. Indeed, Agl15 shares substantial identity (28%) and similarity (51%) with AglR, recently proposed to serve as or to assist the DolP-mannose flippase recruited in the pathway used for pentasaccharide-based glycosylation of S-layer glycoprotein Asn-13 and Asn-83 (Kaminski et al., 2012). Finally, the absence of Agl5–Agl15 did not compromise Asn-13 and Asn-83 glycosylation, arguing that these proteins are dedicated to the assembly of the low salt tetrasaccharide (Kaminski et al., 2013a).

**FIGURE 2:**
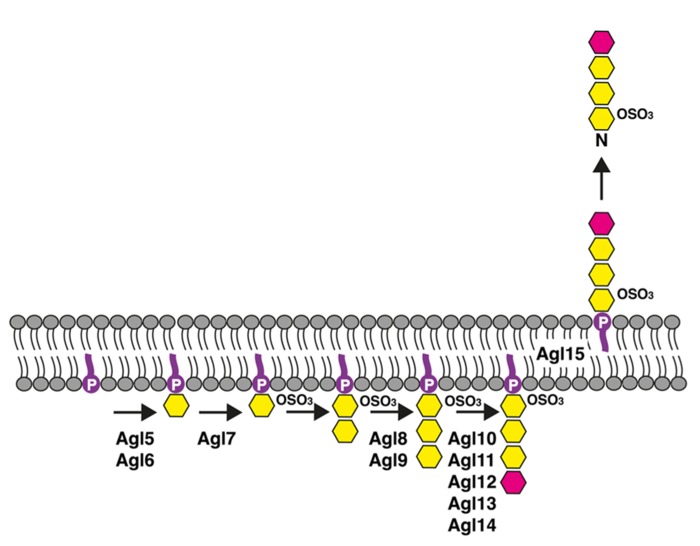
**Schematic depiction of the *Hfx. volcanii* pathway used for the assembly of the tetrasaccharide N-linked to the S-layer glycoprotein Asn-498 position when the cells are grown in low salt (1.75 M NaCl)-containing medium.** See the text for details. In the figure, dolichol phosphate is in purple, hexose is in yellow and rhamnose is in pink. The bottom half of the figure corresponds to the cell interior.

Although *Hfx. volcanii* seemingly relies on two different pathways for the assembly of the two N-linked glycans decorating the S-layer glycoprotein, only one oligosaccharyltransferase, namely the enzyme responsible for the transfer of the lipid-linked glycan to a target protein, has been identified in this organism. In *Hfx. volcanii*, AglB is the only homolog of the eukaryal oligosaccharyltransferase catalytic subunit, Stt3, or its bacterial counterpart, PglB (Magidovich and Eichler, 2009; Kaminski et al., 2013b). As such, the absence of AglB prevented the glycosylation of S-layer glycoprotein Asn-13 and Asn-83 by the pentasaccharide normally attached at these positions (Abu-Qarn et al., 2007). On the other hand, *aglB* deletion had no effect on the appearance of the low salt tetrasaccharide added to the Asn-498 position (Kaminski et al., 2013a). Thus, a currently unidentified and novel oligosaccharyltransferase is seemingly involved in the delivery of the low salt tetrasaccharide (and its precursors) from dolichol phosphate to S-layer glycoprotein Asn-498. The same may be the case in *Hbt. salinarum*, where one of the two N-linked glycans decorating the S-layer glycoprotein in this species is transferred from a dolichol phosphate carrier while the second glycan is delivered from a dolichol pyrophosphate carrier (for review, see Lechner and Wieland, 1989; Cohen-Rosenzweig et al., 2013).

Presently, the reason why the *Hfx. volcanii* S-layer glycoprotein (and the *Hbt. salinarum* S-layer glycoprotein, for that matter) can be modified by two distinct N-linked glycans as a function of environmental salinity can only be supposed. Likewise, the reason why Asn-498 is only modified when cells are grown at a given salt concentration is not clear. One could envisage a salt concentration-related conformational change in the S-layer glycoprotein leading to the exposure of Asn-498 to the low salt tetrasaccharide N-glycosylation machinery only at the lower salinity. Alternatively, modification of Asn-498 could be a question of the availability of the low salt tetrasaccharide since only minute levels of this glycan are bound to dolichol phosphate in cells grown in high salt conditions. Another consideration that requires further study concerns how cells lacking different components of the N-linked pentasaccharide biosynthetic pathway are able to decorate Asn-498 with the low salt tetrasaccharide at elevated salinity (Kaminski et al., 2013a). Finally, as studies on species other than *Hfx. volcanii* begin to provide novel insight into archaeal N-glycosylation, it will be important to determine whether environmental concerns apart from salinity also modulate such protein modification.

## AUTHOR CONTRIBUTIONS

All authors made substantial contributions to the acquisition, analysis, and interpretation of data described in this report. All authors critically reviewed the report and approved the final version. All authors agree to be accountable for all aspects of the work in ensuring that questions related to the accuracy or integrity of any part of the work are appropriately investigated and resolved.

## Conflict of Interest Statement

The authors declare that the research was conducted in the absence of any commercial or financial relationships that could be construed as a potential conflict of interest.
